# Disentangling the Multidimensional Relationship between Livestock Breeds and Ecosystem Services

**DOI:** 10.3390/ani11092548

**Published:** 2021-08-30

**Authors:** Elena Velado-Alonso, Antonio Gómez-Sal, Alberto Bernués, Daniel Martín-Collado

**Affiliations:** 1 Departamento de Ecología Integrativa, Estación Biológica de Doñana (EBD-CSIC), C/Américo Vespucio 26, Isla de la Cartuja, 41092 Sevilla, Spain; 2 GloCEE—Global Change Ecology and Evolution Group, Department of Life Sciences, Universidad de Alcalá, Ctra. Madrid-Barcelona Km 33.600, 28805 Alcalá de Henares, Spain; antonio.gomez@uah.es; 3 FORECO—Forest Ecology and Restoration Group, Department of Life Sciences, Universidad de Alcalá, Ctra. Madrid-Barcelona Km 33.600, 28805 Alcalá de Henares, Spain; 4 Unidad de Producción y Sanidad Animal, Centro de Investigación y Tecnología Agroalimentaria de Aragón (CITA), Avda. Montañana 930, 50059 Zaragoza, Spain; abernues@cita-aragon.es (A.B.); dmartin@cita-aragon.es (D.M.-C.); 5 Instituto Agroalimentario de Aragón—IA2, CITA-Universidad de Zaragoza, Calle Miguel Servet 177, 50013 Zaragoza, Spain

**Keywords:** livestock breed, ecosystem services, biodiversity, livestock systems, nature’s contributions to people, nature-based solutions

## Abstract

**Simple Summary:**

Livestock breeds represent the diversity of livestock animals. They participate in the delivery of ecosystem services (ES), i.e., the benefits to humans provided by nature. In recent years, the contribution of livestock breeds to ES has received attention in livestock research. Additionally, there is increasing interest in integrating this knowledge into policies to make agriculture more sustainable. In this work, we elaborate on livestock breed characteristics that are key to the study of livestock breed contributions to ES. Thus, we explore the natural and human factors that have produced livestock breeds as ecologically and culturally mediated entities. In addition, we review the different roles of livestock breeds as biodiversity components. Finally, we examine how livestock breeds participate in livestock system heterogeneity. By integrating these aspects, we might better understand how livestock breeds provide and modulate ES provision and, therefore, how to improve breed conservation and livestock policies toward more sustainable farming.

**Abstract:**

There is an increasing interest in assessing livestock breed contributions to ecosystem services (ES) and including this knowledge in decision making. However, this task has been limited due to the complexity of the multidimensional relationship between livestock diversity and ecosystem services. In this work, we elaborate on the livestock breed characteristics central to developing a comprehensive approach to livestock breed inclusion in the ecosystem services framework. Thus, we explore the multidimensional nature of livestock breeds, i.e., as eco-cultural entities, biodiversity components, and drivers of livestock system heterogeneity and functioning. First, anthropogenic and natural factors have acted jointly to develop breeds as eco-cultural entities. This fact represents an opportunity to move toward farming system sustainability by Nature-Based Solutions and Nature’s Contribution to People paradigms. Second, livestock breeds are components of biodiversity, and as such, can be framed as goods, as final ecosystem services, and as regulators of ecosystem processes. Third, livestock breeds contribute to livestock system heterogeneity and resilience. By integrating these aspects, we might better understand how livestock breeds provide and modulate ecosystem service provision and, therefore, how to improve breed conservation and livestock policies toward farming system sustainability.

## 1. Introduction

Livestock diversity is increasingly being reported to provide ecosystem services (ES) [1,2]. Ensuring the continued provision of services while reducing disservices is a crucial challenge for the livestock production sector [3,4]. Therefore, there is an emerging interest in identifying and valuing relevant ESs provided by livestock to promote more sustainable food systems [5,6,7,8]. Recently, the contribution of livestock breeds to ES (e.g., [2,5,9,10]) has attracted attention. Nevertheless, this task has been limited since livestock breeds participate in ES provision together with an array of interrelated elements—such as farmers and farms, production systems, and agroecosystems—that complicate this study [11,12].

The ES framework has the potential to reinvigorate interest in livestock breed conservation [8,13], especially in cases of local, rare, or endangered breeds that are usually associated with particular genetic characteristics and singular farming systems. In addition, the ES framework would allow the valuation of distinct breed characteristics, such as adaptation traits and their effects on production systems [14,15]. Furthermore, ES might serve as an indicator or criterion for environmental payments in agricultural and public policies [16]. Therefore, clarifying the relationship between livestock breeds and ES would improve the inclusion of livestock breeds in sustainable management and policy design through specific conservation, environmental and social objectives. In this way, the ES management through livestock breeds could improve the sustainability of livestock systems and agroecosystems [12,17,18] and contribute to biodiversity conservation [19,20].

However, there are some general constraints on putting ES knowledge into practice [21]. The complexity of the livestock breed case makes these constraints even more difficult to overcome. Bennet et al. [22] identified key aspects necessary to incorporate ES research into decision making. First, they proposed a better understanding of the production and provision of ES, considering the wide variety of actors and factors that interact, such as human activities, agricultural practices, scale heterogeneity, or ecological legacy effects. In the case of livestock breeds, this indicated a need for additional research on how and to what extent livestock breeds and their associated actors and factors contribute to different ecosystem services. Second, they found it necessary to identify the diversity of stakeholders who benefit from ES provision and their preferences, ways of use, and supply mechanisms [22]. Regarding livestock breeds, this would involve the diversity of stakeholders participating in their conservation and management at different scales, understanding their context and circumstances. Third, they recommend validating those models and tools by supporting and enhancing better practices, decision-making processes, and more sustainable ES governance [22]. This involves the development of the best local breeds management practices by considering decision-making processes to enhance ES.

To address these questions, some particularities of livestock breeds should be taken into consideration. Livestock breeds are components of biodiversity, with the peculiarity of being eco-cultural entities. Therefore, they are part and drivers of livestock system heterogeneity and functioning. These characteristics determine a plural and complex relationship between ES and livestock breeds, where different perspectives converge. In the following sections, we elaborate on these characteristics focusing on the principal aspects relevant for a comprehensive approach to livestock breed management for decision making under the ES framework.

## 2. Livestock Breed as an Eco-Cultural Entity

Livestock breeds are essentially homogeneous intraspecies populations of domesticated animals with inheritable external traits [23]. However, the breed definition goes beyond a purely genetic definition to a more complex concept, including an essential cultural dimension [24]. Livestock breeds result from coevolutionary processes of livestock populations, management practices, and environmental factors, with an increasing intervention of humans from domestication times to present [25,26]. Within these processes, a large number of factors—e.g., natural selection, artificial selection, history, or geography—have contributed to creating and maintaining a vast variety of livestock breeds over time and space [15,27,28]. For that reason, livestock breeds are conceptualized as eco-cultural—i.e., culturally and environmentally mediated—entities [24,29].

The breed notion is a relatively recent concept. It appeared or was technically formalized in modern times (around the 18th century), connected to the momentum of phenotypic standardization of livestock populations through controlled and systematic selective breeding [26,27,30]. Therefore, the development of standardized breeds in their origin went along with reducing the genetic variability of the original breed populations. Not all livestock populations followed the same selection objectives or experienced the same selection intensity and, ergo, not the same degree of genetic homogenization. Afterwards, the increasing industrialization of agriculture favored a low number of highly productive breeds leading to a sharp reduction in the number of livestock breeds [24,31,32].

Currently, there is international concern about the erosion of livestock diversity [31]. The international community is pursuing conservation [33], which has been targeted as a sustainable development goal [1,34]. Livestock conservation policies have been primarily focused on breeds as genetic resources [29,35]. Nevertheless, the prioritization of livestock breed conservation considering only genetic criteria is somewhat burdensome [36]. Furthermore, if breeds are considered merely genetic resources, their relationship with ES is diluted. Indeed, due to the different determinants of livestock breeds, the conservation of their genetic variability depends on different factors, not only genetic factors (e.g., breeding and genetic conservation programs, in situ or ex situ conservation, gene banks, etc.) but also socioeconomic (e.g., intensification and abandonment of farming, loss of cultural elements linked to farming, etc.) [37].

Therefore, livestock breeds should be considered in their complex biological and cultural position when approaching ES and policy making. Their domesticated nature should be emphasized, and the anthropogenic dimension should be appropriately valued [38]. By framing livestock breeds in that way, we can avoid the tendency to dichotomize humans and nature and find successful transformative solutions to currently entangled environmental and social problems [39]. Consequently, considering the plural meaning of livestock breeds helps to enforce Nature’s Contribution to People paradigm in livestock sciences [40,41]. That is, culture has a central role in sustainable management to tackle contemporary socioenvironmental problems [42].

## 3. Livestock Breeds as a Biodiversity Component

As we have previously explained, livestock breeds are core elements of domesticated diversity, a fundamental part of agrobiodiversity [43]. Thus, just as wildlife biodiversity does, livestock breeds have a multilayered relationship with the hierarchy of ES [44]. They can be conceived as (i) goods, (ii) final ESs, and (iii) a mediator in ecosystem processes. However, the complexity of these three conceptions determines and shapes the case of livestock breed ES.

### 3.1. Livestock Breeds as Goods

Livestock breeds are considered by the World Bank [45] as a global public good, and therefore, according to this institution, their conservation should be a public duty. However, from an economic perspective, they participate in multiple property regimes depending on whether we focus on the individual animals, the herds, or the breed as a biological population. From this perspective, livestock breeds have a triple nature: private goods (i.e., belonging to particular individuals), club goods (i.e., those privately owned but openly used with a shared cost), and commons (i.e., those that could satisfy the whole community) [46].

Hoffmann [46] explored that question further. On the one hand, individual animals and herds are mostly privately owned—individually or collectively—and because of that, they and their genetic improvement, reproduction, and breeding can be understood as pure private goods. On the other hand, breeding livestock is frequently linked to membership in breeding societies. In addition, in certain societies, their products may be labeled under certification schemes. Under these regimes, livestock breeds should also be considered “club goods”. That is, their regulation is controlled by and restricted to members. Furthermore, livestock breeds graze common goods, i.e., they are cultural goods, and they can participate in communal grasslands or be herded within communal pastoral regimes. All of these factors affect how livestock breeds are managed, researched, invested in, and exchanged [46]. Thus, different interests, costs, and benefits arise at different social scales. Farmers are usually interested in productivity and livelihoods, whereas society expects the provision of safe food while maintaining environmental quality [47].

This disparity between private and public, and former, current, and future interests encumbers livestock breed valuation and impacts how ES derived from livestock breeds are perceived and managed. Furthermore, it determines who assumes the costs and consequences (e.g., livestock disservices) of the management and conservation of livestock breeds and the ES linked to them, which to date has not been resolved [24].

### 3.2. Livestock Breeds as Final Ecosystem Services

The final ES are ESs that directly underpin or give rise to a good [44]. Livestock breeds directly provide food, fibers, and other byproducts, transport, and work [1,48]. Moreover, animal genetic resources are utilized in technology and innovation in biotechnology or medicine [49]. Livestock breed diversity maintains and increases the genetic pool and directly fosters the potential for goods and benefits derived from livestock. Additionally, local and rare breeds are specially adapted to specific or extreme environments, linked to local knowledge, and usually provide high-quality products [50]. Accordingly, from the ES perspective, they should be considered strategic as they represent a reserve of scarce genetic traits and adaptations within the whole livestock genetic pool. Therefore, livestock breeds can be considered final ESs following the Mace, Norris, and Fitter proposal [44].

Livestock breeds as animal genetic resources underpin livestock production, adaptation, and selection. Thus, the current and especially future provision of many ESs under global change, directly and indirectly, depends on conserving livestock breeds in their native environment. By making the breed-environment relationship explicit, we open the way for more sustainable production systems with more coherent connections between the environment, local resources, and livestock uses and practices. The separation of breeds from their native environment is a current trend [51]. This decoupling could diminish their ability to cope with a challenging environment. In addition, it could break livestock breeds’ adaptation processes. Additionally, breed performance and reproduction are affected by the environment and its nutritional characteristics, related to differences in breed traits, such as body size or yield potential [52,53,54]. Despite the importance of livestock breed-environment interactions, they have attracted a fair bit of attention from productivity and genetic evaluation perspectives but less so from a conservation and policy-making perspective.

### 3.3. Livestock Breeds as Mediators of Ecosystem Services

Livestock breeds influence the functioning of the agroecosystems [55], as breeds differ in numerous functional traits, including their feeding preferences, metabolism, behavior, etc. [56]. Thus, they channel energy fluxes between trophic levels and participate in nutrient cycles [57,58]. They also influence vegetation structure, affect community dynamics [59,60], and foster landscape complexity [61,62]. Through these processes, livestock breeds influence supporting and regulating ESs. Therefore, breed substitutions in a given agroecosystem would potentially lead to a variation in ES provision. However, the comparative ecology and biogeography of livestock breeds have been largely neglected [51], and the specific role of livestock breeds in the provision of supporting and regulating ESs is not well understood. This is partly because the ecology of livestock breeds intertwines with anthropogenic factors, e.g., those derived from human modification of environments, biotic communities, and inheritance processes, as well as culture and social behavior [38,63]. In addition, the contribution of breeds to the provision and regulation of ES is strongly influenced by the farming system [2,11].

The scientific literature provides some insight into the potential ecosystem regulatory role of livestock breeds. For example, the breed effect on grazing is apparently underexplored in the scientific literature [64]. Some literature seems to point toward minor significance of livestock breeds on grazing [65,66,67]. However, more recent studies have shown the effects of livestock breeds on pasture vegetation composition and plant traits [68,69,70], related to body size, foraging behavior, movements, and specific body traits [56,68]. Other examples are resistance to diseases, greenhouse gas emissions, or habitat provision. Some breeds are recognized as having disease tolerance [29,71]. The most notable case is the N’Dama cattle in Africa [72,73], but other examples have been found in different continents [74]. In the case of nitrogen and greenhouse gases, emission rates by livestock systems depend upon both the animal breeds and diet quality [75]. Despite this, the interaction between different production systems and breeds in driving greenhouse gas emissions has not yet been appropriately studied [76].

In the case of habitat provision, different livestock species and breeds may generate different habitat structures, helping to maintain high natural value habitats [77]. Additionally, some native breeds are specifically adapted to less favorable environments, where usually much wild species biodiversity is found, as these environments work as refuges [78]. Considering a large territorial scale, a positive relationship between the distribution of wild diversity and livestock breed diversity has been found in long-term anthropized areas [20]. More research is needed to understand the livestock breed effect of supporting habitat services for wild species at a specific ecosystem scale [79,80].

The conception of livestock breeds as goods, final ES, and mediators of ecosystem processes determines and enriches the contribution of livestock breeds to ES. However, this conceptual framework is not well recognized or integrated into research and decision making. To date, livestock breed conservation strategies have ranged from production-oriented to service-oriented perspectives [10], but sustainable approaches require more holistic perspectives. In addition, this complexity sometimes leads to a lack of recognition and remuneration of some ES sin livestock systems, especially regulating and cultural ESs [81].

## 4. Livestock Breed as a Driver of Farming System Heterogeneity

Livestock farming systems are highly heterogeneous. Broadly speaking, farming systems can be differentiated according to the movement of animals (i.e., nomadism, transhumance, pastoral, stationary husbandry systems), land use and feeding systems (i.e., landless, mixed systems, grassland-based systems), and the livestock species used or the final products [82]. Furthermore, there is still considerable heterogeneity within and across countries and world regions even within these broad management typologies. All of these characteristics endorse a wide variety of agroecosystems, where different livestock breeds are inherently embedded. In that way, breeds can be seen as a key factor promoting the diversity of livestock production systems and agroecosystems.

Some insights indicate that heterogeneity benefits have been underestimated in livestock systems. These benefits might derive from the positive effects of livestock diversity on farming performance. For example, species, breeds, and management practice diversity might enhance production system performance thanks to complementarity utilizing different grazing resources within the system [17]. Additionally, functional trait diversity within the herd could help to buffer production-efficiency trade-offs [83]. In a similar vein, the diversity of livestock breeds, livestock systems, and agroecosystems can improve the provision of ES and system resilience. For instance, depending on the modality and intensity of the grazing system, different outcomes—positive or negative—appear in the vegetation structure and composition [84,85], nutrient availability [86,87], or landscape configuration [80,88,89]. Thus, livestock system heterogeneity and breed diversity might contribute to livestock system resilience by making the most of less favorable environments.

Therefore, to evaluate the contribution of livestock diversity to ES, studies should include detailed information on livestock type, grazing behavior, timing and frequency of free grazing, duration, and stoking rates [90], which are usually determined by the livestock breed raised. Depending on the case, ES delivery and trade-offs would rely more on the farming system or agroecosystem type than on the specific local breed. Martin-Collado & Bernués [11] defined when to attribute ES delivery to a breed. A direct connection between a breed and a specific ES should only be considered when the delivery of this service relies on the breed, and its replacement significantly alters the farming system and, therefore, the provision of ES.

Livestock systems have rapidly changed over the past decades [91], usually following intensification and homogenization pathways. The most industrialized systems (e.g., landless stationary intensive poultry and pig farms) have led to almost complete disconnection of the farming systems from the ecosystem which holds them [92]. Additionally, these intensive livestock systems usually produce disservices via air, water, and soil pollution due to incorrect waste management and greenhouse gas emissions [93]. Moreover, they favor biodiversity losses in other world regions where farming inputs come from [94]. Both the farming system homogenization process and local breed substitution by transboundary industrial breeds have drastically reduced the range of ES delivered by traditional and multifunctional farming systems, usually based on local breeds. The resulting livestock system heterogeneity markedly determines which and how ESs are delivered or channeled by livestock breeds.

## 5. Final Remarks

**Figure 1:**
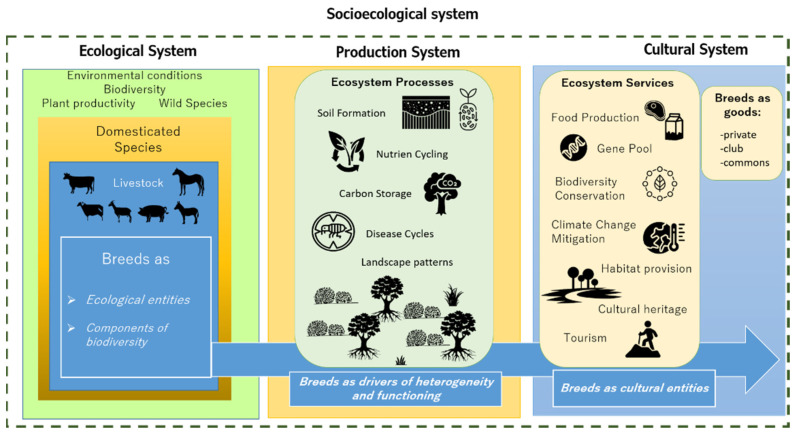
Conceptual framework: livestock breeds as eco-cultural entities, as a component of biodiversity, and as drivers of heterogeneity and the functioning of agroecosystems.

To assess livestock breed contribution to ecosystem services, we consider it necessary to take into account three important aspects. Livestock breeds are eco-cultural entities, biodiversity components, and essential drivers of farming system heterogeneity (the conceptual framework is depicted in Figure 1).

These characteristics must be considered and emphasized when approaching livestock breeds in decision making under the ES framework. First, livestock breeds represent an opportunity to move toward sustainability of human uses and approach Nature’s Contribution to People and Nature-Based Solutions paradigms. Second, by understanding that breeds could be considered private, club, or common goods, we can improve our insights into who benefits from ES provision and how livestock breeds are valued. Third, by considering breeds as final ES and ES mediators embedded in production systems, we can better understand how ES are produced and improve livestock management practices. Acknowledging these factors will help bridge the gap between decision making, livestock breed conservation opportunities, and sustainable ES management.

It should be noted that the relationship between livestock systems and breeds, and their changing nature is a historical process. Likewise, breeds’ relationship with agroecosystems and production systems is dynamic, changing over space and time. This fact determines how ESs derived from livestock systems are perceived and valued.

## Data Availability

Not applicable.
